# Baricitinib Mitigates Methotrexate-Induced Liver Fibrosis Model via YAP Pathway

**DOI:** 10.3390/medicina61050857

**Published:** 2025-05-06

**Authors:** Mehmet Ulusan, Mumin Alper Erdogan, Ozkan Simsek, Zafer Dogan, Bertug Bekir Ciftci, Gultekin Atalan, Oytun Erbas

**Affiliations:** 1 Department of Internal Medicine, Faculty of Veterinary Medicine, Burdur Mehmet Akif Ersoy University, Burdur 15030, Turkey; mehmet.bucak@hotmail.com; 2 Department of Physiology, Faculty of Medicine, Izmir Katip Celebi University, Izmir 35620, Turkey; alpero86@gmail.com; 3 Department of Physiology, Faculty of Veterinary Medicine, Burdur Mehmet Akif Ersoy University, Burdur 15030, Turkey; 4 Department of Surgery, Faculty of Veterinary Medicine, Tekirdag Namik Kemal University, Tekirdag 59010, Turkey; zdogan@nku.edu.tr; 5 Department of Veterinary Surgery, Health Science Institute, Erciyes University, Kayseri 38280, Turkey; 4022740002@erciyes.edu.tr; 6 Department of Surgery, Faculty of Veterinary Medicine, Erciyes University, Kayseri 38280, Turkey; gatalan@erciyes.edu.tr; 7 Faculty of Medicine, BAMER, Biruni University, Istanbul 34015, Turkey; oytunerbas@yahoo.com

**Keywords:** baricitinib, Hippo pathway, liver fibrosis, methotrexate, YAP

## Abstract

*Background and Objectives*: Liver fibrosis, a chronic process caused by various pathogenic factors, including drug toxicity, metabolic disorders, and chronic inflammation, is associated with liver-related mortality rates worldwide. It has been established that methotrexate (MTX), a pharmaceutical agent utilised in the treatment of numerous diseases, induces hepatic fibrosis. Currently, there is still a paucity of clinically efficacious antifibrotic drugs for the management of hepatic fibrosis. Thus, the present research sought to evaluate the antifibrotic effects of baricitinib in a rat model of MTX-induced liver fibrosis through the yes-associated protein (YAP) pathway. *Materials and Methods*: A total of 36 Wistar rats were assigned to three groups (*n* = 12) randomly: a control group, an MTX-induced liver fibrosis group, and a baricitinib-treated group, which received 20 mg/kg/day of baricitinib following fibrosis induction. All treatments were administered for 10 days. *Results*: Biochemical analyses revealed significant increases in plasma alanine aminotransferase (ALT), cytokeratin-18 (CK-18), and malondialdehyde (MDA) levels, as well as liver transforming growth factor-beta (TGF-β), YAP1, and MDA levels, in the MTX-induced fibrosis group in comparison to the control group (*p* < 0.05). Notably, baricitinib addition significantly reduced these biomarkers (*p* < 0.05). A histopathological evaluation further confirmed a marked reduction in fibrosis, hepatic necrosis, and cellular infiltration in the baricitinib-treated group relative to the MTX-induced fibrosis group. *Conclusions*: Accordingly, our findings suggest that baricitinib mitigates MTX-induced liver fibrosis, potentially through its anti-inflammatory and antifibrotic effects mediated by the suppression of the YAP signalling pathway. These results highlight that baricitinib could be a potential treatment option for patients with liver fibrosis.

## 1. Introduction

Liver fibrosis is described as the pathological deposition of the extracellular matrix in hepatic tissue, resulting from a sustained injury–repair process that ultimately disrupts the liver architecture and impairs its activity [1]. The morbidity and mortality of liver fibrosis are significant worldwide, with an increasing prevalence each year [2]. Fibrosis is a characteristic of advanced liver disease and a precursor to severe disorders, including cirrhosis and hepatocellular carcinoma [3].

Methotrexate (MTX), a widely used antifolate drug, is prescribed for the therapy of autoimmune and inflammatory disorders, including psoriasis and rheumatoid arthritis [4,5,6,7]. However, despite its therapeutic benefits, MTX has been associated with hepatotoxicity, and long-term administration, even at low doses, has been reported to induce hepatic fibrosis [8,9,10,11], thus posing a significant clinical challenge. Previous research has suggested that MTX-triggered liver damage is largely driven by oxidative stress, which aggravates liver injury via inflammation, cell death, and mitochondrial dysfunction [12,13,14]. Methotrexate has been shown to inhibit nicotinamide adenosine diphosphate (NADP)-dependent dehydrogenase and malic enzymes, disrupting the cellular redox balance. Nicotinamide adenosine diphosphate is a crucial cytosolic antioxidant required for the activity of glutathione reductase, an enzyme that protects cells from oxidative damage. Consequently, a decline in NADP levels renders hepatocytes vulnerable to injury from reactive oxygen species [8,15,16,17,18]. Numerous drugs are utilised to mitigate MTX-induced hepatotoxicity [19,20,21]; however, their efficacy in preventing hepatic fibrosis remains limited in humans [22,23].

Baricitinib, a targeted Janus kinase (JAK) 1/2 suppressor with potent anti-inflammatory properties, has been authorised for the therapy of rheumatoid arthritis and related inflammatory conditions [24]. Recent studies have demonstrated its efficacy in alleviating fibrosis in pulmonary, dermal, and cardiac tissues [25,26]. Despite these promising findings, the potential antifibrotic effects of baricitinib in liver fibrosis remain unexplored. Considering this, the present research was conducted to investigate the protective role of baricitinib in an MTX-induced hepatic fibrosis model in rats, with a particular focus on its modulation of the YAP signalling pathway.

## 2. Materials and Methods

### 2.1. Animals

The present research utilised thirty-six female Wistar albino rats, with a body mass range of 150–200 g and an age range of 10–12 weeks. Baseline data on the body weights and ages of the rats in each group are presented in Table 1. The animals were procured from the Experimental Animal Centre of Demiroglu Scientific University. The rats were housed in steel cages and provided with unrestricted access to a standard chow diet (comprising 35% fat, 18% protein, and 47% carbohydrates) and water within a temperature-controlled (22 ± 2 °C) environment that was subject to a 12 h light/dark cycle. The experiments conducted in this study were executed in accordance with the guidelines stipulated in the Guide for the Care and Use of Laboratory Animals, as outlined by the National Institutes of Health in the USA. The experiments were conducted only after obtaining ethical permission from the Animal Ethics Committee of Demiroglu Science University (Ethical number: 325011204, 15 January 2023). Unless otherwise stated, all chemicals were supplied by Sigma-Aldrich Inc., Munich, Germany.

### 2.2. Experimental Design

A total of 36 female Wistar rats were randomly allocated to three groups (n = 12). The first group represented the control group and did not undergo any chemical treatment. The second and third group rats were administered a single intraperitoneal (IP) injection of MTX (20 mg/kg) to trigger hepatic injury. Following MTX administration, the third group rats were administered baricitinib (20 mg/kg/day) by IP injection, while the second group was treated with 1 mL/kg/day of 0.9% NaCl saline via the same route. All interventions were administered for a period of 10 days.

Upon completion of the experiment, all animals were euthanised by cervical dislocation following high-dose anaesthesia induced by ketamine (100 mg/kg) and xylazine (50 mg/kg) (Ketasol, Richterpharma AG, Austria; Rompun, Bayer, Germany), in accordance with the AVMA Guidelines for the Euthanasia of Animals. Samples of blood were obtained from the rats through cardiac puncture for a biochemical analysis. The hepatic tissue was then dissected for further assessment by histopathology and biochemistry.

### 2.3. Biochemical Analysis of ALT and CK-18 Levels in Plasma

The measurement of plasma alanine aminotransferase (ALT) and cytokeratin-18 (CK-18) levels was conducted via a commercially sourced ELISA kit provided by USCN Life Science Inc., Wuhan, China.

### 2.4. Lipid Peroxidation Analysis of Plasma

The quantification of lipid peroxidation in tissue and plasma specimens was evaluated by determining malondialdehyde (MDA) concentrations as thiobarbituric acid reactive substances (TBARSs), in accordance with the methodology outlined by Taskin et al. [14]. In summary, trichloroacetic acid and TBARS reagent were introduced to the tissue samples and then mixed and incubated at 100 °C for 60 min. After being chilled on ice, the samples were subjected to centrifugation at 3000 rpm for 20 min. The optical density of the supernatants was then measured at a 535 nm wavelength. The MDA concentrations in the tissue samples were determined through a reference calibration curve prepared with tetraethoxypropane and are presented as nmol per gram of protein.

### 2.5. Biochemical Assessment of TGF-Beta and YAP1 in Liver Tissue

Post-decapitation, the livers were swiftly excised and stored at −20 °C until further analysis. Liver tissue samples were homogenised with a glass homogeniser in a fivefold volume of phosphate-buffered saline adjusted to pH 7.4 for a biochemical analysis. The homogenates were then exposed to centrifugation at 5000 g for a period of 15 min. The resultant clear upper layer was harvested, and the total protein concentration in the liver homogenates was assessed using Bradford’s method, with bovine serum albumin as the reference [27].

The concentrations of transforming growth factor-beta (TGF-β) and yes-associated protein 1 (YAP1) in the liver supernatants were quantified using commercially sourced rat-specific enzyme-linked immunosorbent assay (ELISA) kits (BD Biosciences, San Jose, CA, USA). All samples were analysed in two independent measurements following the manufacturer’s protocols. Absorbance values were obtained via a microplate reader (Multiscan Go, Thermo Fisher Scientific Inc., Portsmouth, NH, USA).

### 2.6. Histological Analysis

Formalin-fixed liver tissue samples (4 μm) were subjected to haematoxylin and eosin staining, and imaging was conducted using an Olympus C-5050 digital imaging system attached to an Olympus BX51 microscope (Olympus Co., Tokyo, Japan).

A Liver histopathological evaluation was performed according to the methodology outlined by Lobenhofer et al. [28]. The evaluation was quantified by summing the individual scores assigned to each liver section parameter (hepatocyte necrosis, fibrosis, and cellular infiltration) using a grading scale of 1 (minimal), 2 (mild), 3 (moderate), and 4 (marked).

### 2.7. Statistical Analysis

Statistical analyses were performed using SPSS version 15.0. Parametric variables were evaluated using Student’s t-test or an analysis of variance (ANOVA), and the results are expressed as mean ± standard deviation (SD). Nonparametric variables were compared using the Mann–Whitney U test. Ordinal data are presented as median (range). Statistically significant results were accepted as those with *p* < 0.05, and markedly statistically significant results were accepted as those with *p* < 0.001.

## 3. Results

### 3.1. Effect of Baricitinib on Biochemical Parameters

In the rats treated with MTX and saline, there was a significant elevation in plasma ALT (*p* < 0.01), CK-18 (*p* < 0.001), and MDA (*p* < 0.001) levels, along with increased hepatic TGF-β (*p* < 0.01), YAP1 (*p* < 0.01), and MDA (*p* < 0.001) levels, in comparison to the control group. However, the administration of baricitinib markedly reduced plasma ALT (*p* < 0.05), CK-18 (*p* < 0.001), and MDA (*p* < 0.05) levels, as well as hepatic TGF-β (*p* < 0.05), YAP1 (*p* < 0.05), and MDA (*p* < 0.001) levels, in the MTX-induced fibrosis group (Table 2).

### 3.2. Effect of Baricitinib on Histopathological Parameters

**Figure 1:**
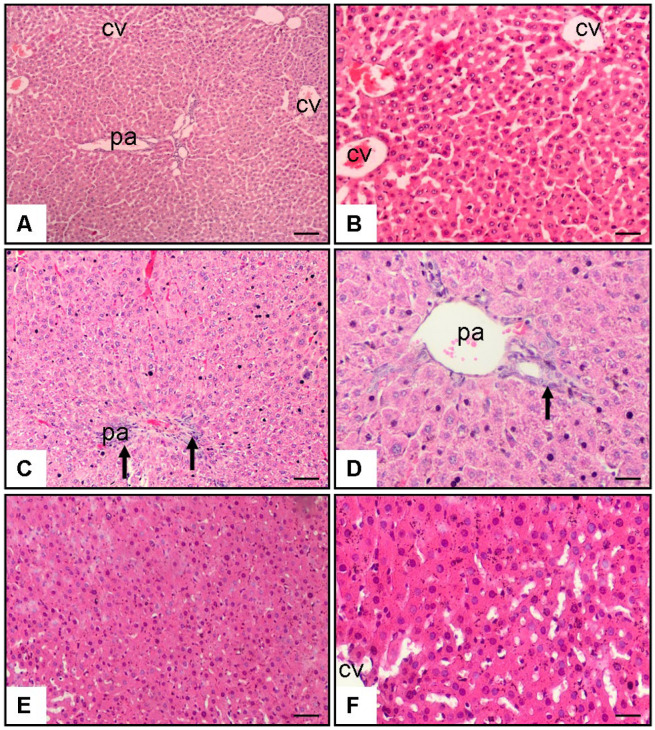
Effect of baricitinib on hepatocytes. Haematoxylin–eosin staining of tissue samples from rat liver (10× and 20× magnification). (**A**,**B**) Control group; CV: central vein; pa: portal area; (**C**,**D**) MTX (20 mg/kg) and saline group (1 mL/kg/day of 0.9% NaCl), arrow indicates reduction in bridging necrosis, fibrosis, and cellular infiltration in pa; (**E**,**F**) MTX (20 mg/kg) and baricitinib (20 mg/kg/day) group.

The histopathological evaluation indicated significant morphological differences among the experimental groups. In the control group, the liver exhibited a normal histological structure with well-organised hepatocytes and intact central veins and portal areas. In contrast, the MTX and saline-treated group demonstrated severe histopathological damage in the portal area, characterised by hepatic necrosis, fibrosis, and marked cellular infiltration. Notably, in the rats treated with MTX + baricitinib, a considerable reduction in these pathological changes was observed (Figure 1).

The histopathological analysis showed that there was a considerable reduction (*p* < 0.01) in liver fibrosis, hepatic necrosis, and cellular infiltration in the baricitinib-supplemented group in comparison to the methotrexate-induced liver fibrosis group (Table 3).

## 4. Discussion

Liver fibrosis is a consequence of advanced hepatic damage caused by various factors, including diseases or side effects of therapeutical drugs, and its prevalence is increasing globally [2,3]. Methotrexate is an agent known to cause histological changes in the liver, including hepatic necrosis, fibrosis, and increased cellular infiltration, as side effects of treating disorders such as psoriasis and rheumatoid arthritis [5,6,7,29,30]. Various pharmacological agents have been utilised to alleviate the adverse effects of MTX [19,20,21]; however, their effectiveness in preventing hepatic fibrosis remains limited in humans [22,23]. Based on this information, the present study investigated the biochemical and histopathological effects of baricitinib, an anti-inflammatory agent, in a methotrexate-induced liver fibrosis model via the modulation of the YAP pathway.

Alanine aminotransferase is a key biomarker of liver injury, reflecting hepatocellular damage and necrosis [31]. In accordance with the findings of earlier studies [30,32,33], we observed a considerable elevation in plasma ALT levels in response to MTX administration, indicative of hepatotoxicity. Importantly, baricitinib supplementation contributed to a considerable reduction in plasma ALT levels, further supporting its protective effects in mitigating MTX-induced hepatic damage.

Cytokeratin-18, a fundamental intermediate filament protein in liver cells, is a primary caspase substrate during hepatic cell death and plays a key role in hepatic injury. Moreover, plasma CK-18 is highly specific for detecting fibrosis [34]. Consistent with this, rats with MTX-induced liver fibrosis were found to have increased CK-18 levels in the present study. The addition of baricitinib reduced the CK-18 levels in these fibrosis-induced rats, suggesting that baricitinib may exert hepatoprotective effects by attenuating hepatocyte apoptosis and preserving liver integrity.

As shown in previous studies [35,36,37], MTX administration elevates serum and tissue MDA levels in rats, a key marker of lipid peroxidation and oxidative stress. Consistent with these findings, our study demonstrated a considerable elevation in MDA levels in both plasma and liver tissue following MTX treatment in comparison to the control group. However, baricitinib administration effectively reduced MDA levels, indicating a potential antioxidant role in mitigating oxidative stress-associated liver injury.

Transforming growth factor-beta is a key fibrogenic cytokine involved in the progression of chronic liver disease, contributing to fibrosis development by promoting hepatic stellate cell activation and extracellular matrix deposition [38]. Our results demonstrated that MTX treatment led to elevated liver TGF-β levels, further confirming its role in fibrosis progression. However, baricitinib supplementation significantly suppressed TGF-β expression, suggesting its potential antifibrotic activity, likely through the modulation of inflammatory and fibrotic signalling pathways.

Recent studies have emphasised the function of the Hippo pathway in fibrosis in different tissues, notably in the lung [39], liver [40,41,42,43,44], kidney [45], and heart [46]. Yes-associated protein 1 is a key mediator of the Hippo pathway and plays a crucial role in hepatic stellate cell stimulation, a key process in the initiation and progression of hepatic fibrosis [47]. Consistent with these findings, this study demonstrated that MTX-induced liver fibrosis was related to elevated liver YAP1 expression. Notably, baricitinib supplementation reduced YAP1 levels in fibrotic rats, suggesting that its antifibrotic effects may be mediated via the inhibition of the YAP1 pathway.

A histopathological analysis showed that there was a significant reduction in liver fibrosis, hepatic necrosis, and cellular infiltration in the baricitinib-supplemented group in comparison to the methotrexate-induced liver fibrosis group. The presence of bridging necrosis and fibrosis in the MTX-treated group aligns with previous research [13,14] reporting MTX-induced hepatotoxicity. MTX is known to induce liver injury through oxidative stress and inflammatory pathways [36,37]. In our study, the attenuation of fibrosis and necrosis in the baricitinib-treated group suggests a potential hepatoprotective role of this drug, likely mediated through its anti-inflammatory and antifibrotic properties. By inhibiting the JAK/STAT signalling pathway, baricitinib may suppress pro-inflammatory cytokine activity, thereby reducing tissue damage. However, further biochemical and molecular analyses are warranted to elucidate the precise processes responsible for the hepatoprotective effects of baricitinib.

## 5. Conclusions

In conclusion, this research revealed the biochemical and histopathological advantages of oral baricitinib administration in a model of MTX-induced hepatic fibrosis in rats. The findings indicate that baricitinib reduced plasma ALT, CK-18, and MDA levels, as well as liver TGF-β, YAP1, and MDA levels. Moreover, a histological analysis verified a considerable decline in hepatic necrosis, fibrosis, and cellular infiltration. Collectively, these observations suggest that baricitinib provides partial protection against MTX-induced hepatic injury, indicating its potential relevance in future therapeutic approaches. However, further studies are required to validate the findings and investigate the fundamental molecular pathways of baricitinib in liver fibrosis.

## Figures and Tables

**Table 1:** Baseline body weights and ages of rats in each group (mean ± SD).

Group	Body Weight (g)	Age (Weeks)
Control	176.5 ± 9.4	11.2 ± 0.4
MTX + Saline	178.1 ± 10.2	11.3 ± 0.5
MTX + Baricitinib	177.3 ± 8.9	11.1 ± 0.6

**Table 2:** Effects of baricitinib on plasma and liver biochemical markers in rats.

	Control	MTX + Saline	MTX + Baricitinib
Liver TGF-β level (pg/g)	0.58 ± 0.2	2.6 ± 0.1 *	1.15 ± 0.1 #
Liver MDA level (nmol/g tissue)	30.8 ± 0.6	68.2 ± 3.5 **	45.4 ± 1.8 ##
Liver YAP1 level (pg/g)	635.1 ± 4.6	958.2 ± 2.4 *	715.9 ± 1.8 #
Plasma cytokeratin-18 level (ng/mL)	1.2 ± 0.1	2.75 ± 0.6 **	1.9 ± 0.3 ##
Plasma MDA level (nM)	32.5 ± 1.8	122.4 ± 6.5 **	106.8 ± 3.1 #
Plasma ALT (IU/L)	16.5 ± 0.8	44.2 ± 1.5 *	32.3 ± 1.6 #

Data are presented as mean ± SD. Control group: standard chow diet; MTX + saline group: MTX (20 mg/kg) and saline (1 mL/kg/day of 0.9% NaCl); MTX + baricitinib group: MTX (20 mg/kg) and baricitinib (20 mg/kg/day). * *p* < 0.01 and ** *p* < 0.001 indicate differences from the control group; # *p* < 0.05 and ## *p* < 0.001 indicate differences from the MTX + saline-treated group.

**Table 3:** Effect of baricitinib on morphometric histological grading of hepatic necrosis, fibrosis, and cellular infiltration.

	Control	MTX + Saline	MTX + Baricitinib
Hepatic necrosis	0 [0–1]	2 [2,3] *	1 [0–2] #
Fibrosis	0 [0–1]	2 [1,2,3] *	1 [0–2] #
Cellular infiltration	0 [0–1]	2 [1,2,3] *	1 [0–2] #

Data are expressed as median [minimum–maximum]. Control group: standard chow diet; MTX + saline group: MTX (20 mg/kg) and saline (1 mL/kg/day of 0.9% NaCl); MTX + baricitinib group: MTX (20 mg/kg) and baricitinib (20 mg/kg/day). * *p* < 0.001 indicates considerable differences from the control group; # *p* < 0.001 denotes considerable differences from the MTX + saline group.

## Data Availability

All data obtained from this study are included in this article.
